# Prevention of fatigue and insomnia in shift workers—a review of non-pharmacological measures

**DOI:** 10.1186/s13167-016-0064-4

**Published:** 2016-08-02

**Authors:** Kneginja Richter, Jens Acker, Sophia Adam, Guenter Niklewski

**Affiliations:** 1 Center for Sleep Medicine, University Clinic for Psychiatry and Psychotherapy, Paracelsus Medical University Nuremberg, Prof.-Ernst-Nathan-Straße 1, D-90419 Nuremberg, Germany; 2 Faculty for Social Sciences, Georg Simon Ohm University for Applied Sciences, Nuremberg, Germany; 3 Medical Faculty, University of Goce Delcev, Stip, Macedonia; 4 Clinic for Sleep Medicine, Bad Zurzach, Switzerland; 5 Department of Psychology, Faculty of Humanities, Social Sciences, and Theology, Friedrich-Alexander University Erlangen-Nuremberg, Erlangen, Germany

**Keywords:** Predictive preventive personalized medicine, Shift work, Socioeconomic factors, Mood disorders, Innovative strategies, Risk factors, Chronic fatigue, Comorbid insomnia, Health promotion, Work environment

## Abstract

**Background:**

Excessive fatigue and insomnia are common among shift workers and can lead to negative effects such as reduced work performance, processing errors, accidents at work, absenteeism, reduced quality of life, and symptoms of depression. Moreover, work in rotating shifts can be a risk factor for different somatic and psychiatric diseases and may contribute to poor health, especially in elder adults and women. This review aims to show non-pharmacological preventive measures against fatigue and insomnia in shift workers.

**Method:**

Computerized literature searches in MedLine and in the Cochrane Library were performed with the following key words: shift work disorder, fatigue, insomnia, shift work, measures, treatment, therapy, strategies and coping. The search was limited to non-pharmacological studies that were conducted on human subjects and published as English-language articles in peer-reviewed journals since 1970. Additional studies were identified through the reference sections of relevant articles. Eighteen articles on fatigue in shift workers, including six original research articles with a total sample size of 3504 probands consisting of industrial workers, office employees, aircraft maintenance engineers, and non-shift workers working in simulated shifts, were analyzed, as well as seven articles on insomnia, including an original research article with a sample size of 26 media workers. Also, 4 reviews on shift work disorder were analyzed.

**Main:**

The occurrence of fatigue and insomnia in shift workers associated with a working period is described as shift work disorder. Estimations on the prevalence of shift work disorder in shift workers vary between 5 % and about 20 %; about one in three shift workers is affected by insomnia and up to 90 % of shift workers report regular fatigue and sleepiness at the workplace. We concluded that there is a necessity for treatments to improve the sleep quality of the shift working population. The most common non-pharmacological recommendations to improve sleep quality and to reduce insomnia and fatigue were scheduling, bright light exposure, napping, psychoeducation for sleep hygiene, and cognitive-behavioral measures.

**Conclusion:**

Some important preventive coping strategies for fatigue associated with shift work such as napping and exposure to bright light have already been investigated and are generally approved. A few studies also provide good evidence for the efficacy of cognitive-behavioral techniques in the treatment of chronic primary and comorbid insomnia. These coping strategies summarized in this paper should be considered in the workplace health promotion programs of each work environment to improve working conditions for shift workers and to save money.

## Background

The desynchronization of the biological clock caused by shift work can lead to physical and psychiatric diseases. Fatigue and insomnia resulting from a disturbed sleep-wake cycle occur frequently in shift workers and may manifest as the so-called shift work disorder (SWD, 2016 ICD-10-CM: G47.26) [1–8].

According to the ICSD 2 classification, essential symptoms of shift work sleep disorder are insomnia or excessive sleepiness that occurs as transient phenomena in relation to work schedules. Different schedules belong to the term “shift work”-occasional overnight duty, rotating shifts, permanent night shifts, or early morning work (starting between 4 and 7 a.m.) [4].

The condition usually persists for the duration of the work-shift period. Shift work is common in protective services, transportation, healthcare, and other services that directly affect the health and safety of other [9]. The consequences of shift work disorder can negative affect the physical and mental health, quality of life, productivity, and work performance as well [7, 10–15]. Shift workers diagnosed with SWD usually have a primary complaint of insomnia or excessive sleepiness; the primary complaint is temporally associated with a work period that occurs during the habitual sleep phase. A loss of the normal sleep-wake pattern usually appearing in SWD patients can be shown with polysomnographics or the Multiple Sleep Latency Test [4]. Drake, Roehrs, Richardson, Walsh, and Roth investigated 2570 shift working individuals and reported differences in the prevalence of SWD in night shift workers and rotating shift workers. Fourteen percent of the night workers and 8 % of the workers on rotating shifts met criteria for SWD [5]. Among workers on an oil rig, for example, the prevalence of SWD was estimated at about 23 % [16]. The American Academy of Sleep Medicine estimates the prevalence of SWD, depending on which country is considered, between 5 and 8 %, with night shift workers being affected particularly frequently [4]. Thus, not every shift worker will develop physical or psychiatric diseases but the prevalence is quite high. SWD is assumed to be caused by a misalignment of the circadian rhythm and environmental stimuli and may have potentially harmful consequences on an individual's health status and quality of life [1, 4, 17]. Waage and colleagues showed in a longitudinal study including 1533 nurses important risk factors for SWD: the numbers of night shifts worked in the previous year, composite score on the Epworth Sleepiness Scale, use of melatonin, use of bright light therapy, and symptoms of depression [18]. Another risk factor pointed out by Kalmbach and colleagues is working in rotating shifts. In the sample, odds were over five times greater for highly sleep-reactive individuals to develop SWD after transitioning to rotating shifts [19].

Vallieres and colleagues investigated the sleep pattern of 418 adults, including 51 night workers, 158 rotating shift workers, and 209 day workers, reported night and rotating shift workers with insomnia to have a sleep profile similar to that of day workers with insomnia [20].

Moreover, a positive association between shift work, insomnia, and fatigue has been shown [21]. Insomnia (2016 ICD-10-CM: F51.0) is one of the core symptoms of SWD. The prevalence is about 6 % in the total population [22] and affects between 29 and 38 % of the shift workers [23]. Symptoms include difficulties falling asleep, a disturbed sleep duration, frequent awakening, and an intense focus on the sleeping behavior itself [10, 24, 25]. The socioeconomic and health-related consequences of insomnia in shift workers such as absenteeism and mood disorders [26, 27] should likewise not be underestimated.

The same applies to fatigue (2016 ICD-10-CM: R53). Core symptoms of fatigue are exhaustion, tiredness, and lethargy. The effects of fatigue are also undesirable and cause a loss of efficiency, concentration difficulties, as well as decreased productivity and safety at work [28]. Up to 90 % of the shift workers report sleepiness and fatigue at work [29]. Long and erratic working hours can lead to acute and chronic sleep deprivation as well as a poor sleep quality and thus result in impairments in several domains, including attention, cognition, and motor skills [30]. Also, rotating and night shifts can be a risk factor for chronic and cardiovascular diseases [31, 32], digestive diseases [33], metabolic diseases [34], and diabetes [35].

Many authors have researched the risks that result from shift work, long working hours [36], and a short sleep duration [37, 38]; they found that shift work increases the risk for attention deficits [39] and accidents at work [40–42]. The risk potential of night (28 %) and evening shifts (15 %) is here higher than that of day shifts [43].

For women, there is a high risk of negative health consequences from shift work - especially women over 50 often suffer from fatigue, and female night shift workers have a 28 % risk for breast cancer [44, 45]. Women who have been working night shifts for more than 20 years have an even further increased risk for breast cancer [46, 47]. There are some references providing evidence that night and shift work increase the risk of ovarian [48], colorectal [49], prostate [50], and endometrial cancer [51]. The health risks presented here are well-known health risks encountered by shift workers. Since approximately 15 to 26 % of the US labor force work night, evening, or rotating shifts [52], there is an urgent need for coping strategies in the workplace.

## Review

### Methods

We conducted three separate computerized literature searches in MedLine and in the Cochrane Library. The following key words in different combinations were used to search for the theme of *fatigue*: fatigue and shift work, measures, treatment, therapy, strategies, and coping. For *insomnia*, we searched for insomnia and shift work, measures, treatment, therapy, strategies, and coping. The following key words in different combinations were used to search for articles regarding *shift work disorder*: measures, treatment, therapy, strategies and coping. We ordered the articles from these searches into the categories fatigue and insomnia. In the process, we focused on articles that were based on research on human subjects and published in peer-reviewed journals between 1970 and 2015. Pharmacological studies were excluded, i.e., any study that involved treatment with drugs was not included in the analysis; in other words, only interventions with the intent to improve health and well-being that did not involve the use of any drug or medicine, e.g., psychoeducation on sleep hygiene, cognitive-behavioral techniques, or changes in light exposure, were included. In addition to the results of these searches, reference sections of relevant articles were used to identify further studies that had not come up in the database searches.

On the topic of insomnia in shift work, the electronic databases cited 60 articles. After excluding papers that did not meet the criteria, eight papers that strictly focused on the relevant topics were left and included in our study. On the topic of fatigue in shift work, 79 papers were found and reviewed, of which 19 papers met the inclusion criteria.

### Results

In the following, some generally acknowledged coping strategies against fatigue and insomnia in shift workers are presented: napping, nutrition, light, scheduling, lifestyle training, and cognitive and behavioral interventions. We were able to find that these measures are common practice and scientifically sound. While there is plenty of research on fatigue, there are evidently fewer studies dealing with measures against insomnia in shift workers. For an overview of measures against fatigue, see Table 1 [13–15, 53–67], and against insomnia, see Table 2 [15, 59, 68–72]. For an overview of measures against SWD, see Table 3 [9, 46, 73, 74].

**Table 1:** Recommendations for measures to prevent fatigue in shift workers

Authors	Citation	Title	Type of paper	Recommendations	Sample size	Sample occupation	Population background
Purnell, Feyer, and Herbison	[53]	The impact of a nap opportunity during the night shift on the performance and alertness of 12-h shift workers	Original	Nap	24	Aircraft maintenance engineers working in 12-h rotating shifts	Age 21–59, all male, at least 4 months of experience
Morgenthaler et al.	[13]	Practice parameters for the clinical evaluation and treatment of circadian rhythm sleep disorders. An American Academy of Sleep Medicine report	Systematic review	Nap			
Hajak	[54]	Sleepless in a 24-h society. When inner and external rhythms collide	Systematic review	Scheduling, light exposure, consideration of suitability for shift work			
Juda, Vetter, and Roenneberg	[55]	Chronotype modulates sleep duration, sleep quality, and social jet lag in shift workers	Original	Scheduling	238	Full-time workers in rotating shifts	83 women, 155 men; average age 38.8 ± 9.6 years
Härmä, Tenkanen, Sjoblom, Alikoski, and Heinsalmi	[56]	Combined effects of shift work and lifestyle on the prevalence of insomnia, sleep deprivation and daytime sleepiness	Original	Regular exercises, but not right before an evening shift	3020	Employees of postal and telecommunication agencies, the railway company, and 5 industrial companies, workers in the forest industry and heavy workers	All male, 45–60 years
Roth	[57]	Appropriate therapeutic selection for patients with shift work disorder	Guideline	Timed light exposure			
Kolla and Auger	[58]	Jet lag and shift work sleep disorders: how to help reset the internal clock	Systematic review	Exposure to bright light			
Thorpy	[14]	Understanding and diagnosing shift work disorder	Systematic review	Exposure to light			
Thorpy	[15]	Managing the patient with shift work disorder	Overview	Behavioral measures (exercise and improved sleep hygiene)			
Barion and Zee	[59]	A clinical approach to circadian rhythm sleep disorders	Review	Combination of sleep hygiene education and timed exposure to bright light			
Zisapel	[60]	Circadian rhythm sleep disorders: pathophysiology and potential approaches to management	Review	Bright light/avoidance of light at different times			
Aelfers, Bosma, Houkes, and van Eijk	[61]	Effectiveness of a minimal psychological intervention to reduce mild to moderate depressions and chronic fatigue in a working population: the design of a randomized controlled trial	Two-armed-randomized controlled trial	MPI (minimal psychological intervention)	124	Workers suffering from (chronic) mental fatigue or mild to moderate depression	115 men and 9 women
Schaefer, Williams, and Zee	[62]	Sleep and circadian misalignment for the hospitalist: a review	Review	Sleep hygiene, caffeine, bright light exposure, and planned naps			
Paul, Miller, Gray, Buick, Blazeski, and Arendt	[63]	Circadian phase delay induced by phototherapeutic devices	Original	Bright light exposure	14		7 men, 7 women age: 18–51
Beaumont et al.	[64]	Slow release caffeine and prolonged (64 h) continuous wakefulness: effects on vigilance and cognitive performance	Original	Caffeine	17	Healthy male volunteers	All male from 19–27, mean age: 23 ± 2 years
Crowley, Lee, Tseng, Fogg, and Eastman	[65]	Combinations of bright light, scheduled dark, sunglasses, and melatonin to facilitate circadian entrainment to night shift work	Original	Bright light and dark sunglasses	67	Non-shift workers worked in 5 consecutive simulated night shifts	35 females, 32 males between 18 and 43 (23.9 ± 6.2)
Eastman	[66]	Circadian rhythms and bright light: recommendations for shift work	Review	Light-work-sleep schedules			
Lowden, Moreno, Holmbäck, Lennernäs, and Tucker	[67]	Eating and shift work—effects on habits, metabolism, and performance	Review	Wholefood diet and regular food intake

**Table 2:** Recommendations for measures against insomnia among shift workers

Authors	Citation	Title	Type of paper	Recommendations	Sample size	Sample occupation	Population background
Penn and Bootzin	[68]	Behavioral techniques for enhancing alertness and performance in shift work	Systematic review	Work-related factors: rest periods, variation, complexity and interest value of work, self-pacing of work, feedback/knowledge of results, incentives. Sensory and kinesthetic stimulation, physiological regulation, cognitive skills. Interventions to affect sleep, stress, and family life			
Kerin and Aguirre	[69]	Improving health safety, and profits in extended hours operations (shift work)	Overview	On-the-job training, scheduling, and involvement of the family			
Neil-Sztramko, Pahwa, Demers, Gotay	[70]	Health-related interventions among night shift workers: a critical review of the literature	Systematic review	Controlled light exposure, shift schedule, behavioral and pharmacological interventions			
Järnefelt, Lagerstedt, Kajaste, Sallinen, Savolainen, and Hublin	[71]	Cognitive-behavioral therapy for shift workers with chronic insomnia	Non-randomized intervention	Cognitive-behavioral therapy	26 (19 included in analysis)	Media workers from a broadcasting company diagnosed with insomnia	13 males and 13 females, age 43.5 ± 8.4
Thorpy	[15]	Managing the patient with shift work disorder	Overview	Behavioral measures (exercise and improved sleep hygiene)			
Barion and Zee	[59]	A clinical approach to circadian rhythm sleep disorders	Review	Combination of sleep hygiene education and timed exposure to bright light			
Rajaratnam, Howard, and Grunstein	[72]	Sleep loss and circadian disruption in shift work: health burden and management	Systematic review	Nap

**Table 3:** Recommendations for measures against insomnia among shift workers

Authors	Citation	Title	Type of paper	Recommendations	Sample size	Sample occupation	Population background
Wright, Bogan and Wyatt	[9]	Shift work and the assessment and management of shift work disorder	Review	Approaches to promote sleep, wakefulness, and adaptation of the circadian clock to the imposed work schedule.			
Zee and Goldstein	[73]	Treatment of shift work disorder and jet lag	Review	Temporary bright light exposure during the shift, avoidance of bright light toward the latter portion of the work period, adequate opportunity for sleep			
Bajraktarov, Novotni, Manusheva, Nikovska, Miceva-Velickovska, Zdraveska, et al.	[46]	Main effects of sleep disorders related to shift work-opportunities for preventive programs	Review	Physical fitness, sleep hygiene, timed exposure to light			
Eastman, Boulos, Terman, Campbell, Dijk, Lewy	[74]	Light treatment for sleep disorders: consensus report. VI. Shift work	Review	Timed exposure to light			

Neil-Sztramko, Pahwa, Demers, and Gotay provided an extensive review on health-related interventions among shift workers. All studies were conducted with participants working night shifts. Because of the heterogeneity regarding study samples, jobs, types of intervention, outcomes, results, and study quality, no estimation of effect sizes could be made. Moreover, different chronotypes were not taken into consideration and there were few age-specific analyses. Besides that, due to the setting at the workplace, studies were often neither randomized nor blinded. Still, four types of interventions could be identified: controlled light exposure, shift schedule adjustments, behavioral interventions, and pharmacological interventions. These findings indicate that there is no unrestrictedly useful treatment since individuals respond differently to each intervention. Rather, tailored and combined interventions and holistic approaches should be targeted [70].

In some cases, medical treatment should be recommended in addition to non-pharmacological interventions. Hajak and Zulley analyzed SWD in a systematic review and recommended optimizing the shift plan, enhancing concentration through light exposure, observing sleep hygiene, and, in some cases, medical treatment. It should be noted that only people with the full spectrum of shift work disorder should get medical treatment and that medication does not alleviate the underlying cause, i.e., the disturbance of the circadian rhythm [54].

#### Napping

Short naps which minimize sleep inertia can generally be regarded as helpful [72, 75]. Purnell and colleagues examined 24 engineers working for an airline company in a crossover-designed study. During 2 weeks, they examined the influence of a 20-min nap in the workplace between 1 and 3 a.m. In the first week, employees took naps during their shifts; whereas in the second week, no nap was taken. They found that during the week with naps, employees performed better in a vigilance test (reported *p* < 0.01), suggesting that they were more alert in the workplace and that they could sleep well at home [53]. Morgenthaler et al. recommend a 20-min nap during working hours and exposure to light for 4 × 20 min immediately after the beginning of the shift or, at the latest, before half of the work period is over [13]. According to some more consistent findings in several laboratory simulations and field investigations, short naps improve alertness, vigilance, reaction times, and performance and decrease the risk of accidents [76]. Rajaratnam, Howard, and Grunstein give the general advice that a 20–30-min nap during the night shift or napping for periods between 30 min and 2 h before evening or night shifts can help to maintain alertness and wakefulness [72]. However, napping itself leads to sleep inertia and may even impair vigilance. Hence, the efficacy of napping depends mostly on the timing and duration as well as on the stage of sleep and the particular circadian phase.

#### Nutrition

Some studies also recommend the use of caffeine, especially at the start of a night shift [62, 77]. Beaumont and colleagues provided evidence for the positive effects of caffeine on vigilance (reported *p* < 0.01) and cognitive performance (reported *p* < 0.05) during a long wakefulness period [65]. Nutrition is also an important factor for employees working shifts. Paz and Berry investigated the beneficiary influence of certain meal compositions on the performance of shift workers. They showed that the ratio of protein and carbohydrates should be 1/3 to provide an optimal diet for shift workers. Differences in psychometric performance were correlated with glucose (reported *p* = 0.05) and insulin concentrations (reported *p* = 0.04), and a large neutral amino acid ratio was correlated with alertness (reported *p* = 0.05) [78]. Lowden and colleagues [67] reviewed the influence of nutrition and specific meal patterns on shift workers’ performance. They provided some useful nutrition guidelines for shift workers, yet individual metabolic differences and the willingness to adapt eating habits to the shift schedule should be taken into consideration. A wholefood diet consisting of vegetables, fruit, wholegrain, and low-carb food such as cottage cheese and eggs, and the avoidance of sugar-rich products with a high glycemic load and convenience foods, is advised. Despite the night work, regular normal day and night eating patterns should be maintained.

#### Light

Shift workers experience a circadian misalignment since they have to work when their bodies are actually prepared to sleep. Melatonin is suppressed by (indoor) light. As a result, suppressed endogenous levels of melatonin were found in employees working night shifts [79]. However, the exposure to bright artificial light can counteract this suppression and may lead to the circadian adaptation to night or shift work [80].

As Eastman mentions in his review, the efficacy of treatments with bright light depends on many variables such as the intensity, scheduling, and surrounding of the procedure. Moreover, he points out aspects of feasibility since individual light-work-sleep schedules need to be supported by the company [66]. Still, there is well-founded evidence underlining the benefits of a scheduled exposure to light [81]. In many cases, bright light reduces sleepiness, improves alertness, and leads to better physical fitness, a more balanced sleep pattern, and higher efficiency. As Paul et al. showed, light tower use between 00.00 and 02.00 a.m. causes melatonin suppression and thus diminishes sleepiness in the work place (reported *p* < 0.003) [63]. Even a delayed sleep phase syndrome can be counteracted by exposure to bright light in the early morning and avoidance of light in the evening. With this measure, a phase shift may be effected. Exposure to bright light in the evening may be effective in delaying the internal clock and can be effective against advanced sleep phase syndrome [60]. For the planned exposure to bright light, the recommendations of the American Academy for Sleep Medicine are considered the gold standard. These guidelines also recommend planned light exposure in the work environment and light restriction in the morning [13]. Another recommendation is reversely dealing with light. The recommendation here is to wear sunglasses or even blue blocking glasses before leaving the workplace at the end of the shift or on the way back home [65]. Nonetheless, this advice should be treated with caution because dark glasses may reduce reaction times when driving and thus potentially cause car accidents.

#### Scheduling

In a naturalistic study by Juda, Vetter, and Roenneberg, shift workers were asked to complete the Munich ChronoType Questionnaire for shift workers (MCTQShift) and the Sleep Questionnaire from the Standard Shiftwork Index (SSI). The results (length of sleep, social jet leg, and sleep disturbances) were modulated by the respective chronotype: e.g., “early-birds” had a shorter sleep duration (reported *p* < 0.001) and a more intense social jet lag (reported *p* < 0.001) on night shifts than the “night-owls.” Therefore, it is recommended to take the particular chronotype into consideration when assigning the day, night, or rotating shift to individual employees [55].

#### Lifestyle training

Lifestyle training is a measure that goes one step further: it provides a holistic approach using counseling and psychoeducation [82]. Circadian Technologies, Inc. (CIRCADIAN) is a company that helps employees to cope with the challenges of shift work. Amongst other companies, CIRCADIAN designed the *Managing a Shiftwork Lifestyle* program, an on-the-job training program to support employees and their families with issues associated with shift work. Kerin and Aguirre [69] tested the *Managing a Shiftwork Lifestyle* intervention. Three-hundred thirty mine workers attended a 4-h training workshop in groups of 10 to 50 people. Before the training started, information about the employees’ sleeping habits were assessed and collected over a period of 28 days. Pre- and post-implementation data were compared to determine the net impact of the training, which included information on healthy nutrition, managing fatigue and alertness levels, advice on how to sleep better, recommendations for using naps effectively and on balancing work and home life. Many positive changes could be noted. After the training, 77 % of the participants described their general state of health as “good,” compared to 59 % before the training. Especially gastrointestinal diseases were significantly alleviated. Furthermore, participants spent more time in bed. After the intervention, they, on average, rested for 5.8 h compared to 4.8 h before the intervention and their coffee consumption decreased. More than half of the participants recognized changes in their sleeping environments and, in addition to that, the improvement of the sleep quality correlated with higher alertness and safety. Results were quite positive and emphasize the need for specialized training and education for shift workers. The intervention was also economically successful: it led to lower levels of turnover and, as a result, the employer saved $952 per worker. Additionally, $940 per worker was saved by reducing absenteeism caused by illness. In total, the annual savings amounted to $1892 per employee. However, only few companies involved the families of the shift workers in the training although it can be very effective to give them a better understanding of how to adjust to a shift worker’s schedule with respect to the need for sleep, healthy nutrition, and the organization of family activities [1, 69].

#### Cognitive and behavioral interventions

Cognitive and behavioral interventions can also be useful in coping with the effects of shift work [65]. Behavioral measures beyond improved sleep hygiene, such as exercise, can enhance sleep quality and combat insomnia and excessive sleepiness [53]. Most effective are the optimal timing of work breaks, social activities during breaks, and sensory stimulation, but those techniques still have to be evaluated within a shift work setting [62]. The post-intervention results of a study by Järnefeld and colleagues [71] showed significant improvements in self-reported (reported *p* = 0.002) and actigraphic (reported *p* < 0.05) sleep onset latency, self-reported sleep efficiency (reported *p* = 0.006), self-reported sleep quality (reported *p* < 0.001), and self-reported restedness (reported *p* < 0.05) through cognitive-behavioral strategies. In addition, the perceived severity of insomnia, sleep-related dysfunctional cognitions, psychiatric and somatic symptoms, and the mental component of health-related quality of life improved significantly. The improvements due to the cognitive-behavioral interventions were long-lasting and even increased over the follow-up period. In general, participants had a significantly better sleep quality on days off than on work days, but the treatment still improved their sleep quality at any point in time.

## Conclusions

Unhealthy working conditions may cause illness in employees if they permanently have to work under harmful conditions. Guidelines such as the “Luxembourg Declaration on Workplace Health Promotion in the European Union” contain rules of conduct for employees and employers. Employers should reduce risk factors at work and strengthen protective factors which help to improve or maintain the health of their employees [83]. Workplace health promotion should uncover health-related risk factors and inform employees about the link between lifestyle and the risk of disease in detail. This in particular affects shift workers who suffer from fatigue and insomnia as part of a shift work disorder [5], because workers who suffer from fatigue and insomnia often have no choice but to work when tired. Since insomnia can lead to depression and metabolic diseases, effectively coping with insomnia can help to protect mental and physical health [84]. Insomnia and fatigue may also have severe consequences such as reduced work efficiency, work accidents caused by reduced concentration, and absenteeism [27]. These consequences of overfatigue are last but not least accompanied by financial costs and should therefore be avoided.

The main reasons for fatigue associated with shift work are the disturbance of the circadian rhythm, irregular interruptions of the sleep-wake cycle, and sleep loss [85], accompanied by other contributing factors such as lifestyle, anxiety, or mood disorders [86]. Both specific professional groups and females (typically nurses) are particularly affected by the effects of shift work and therefore need special attention [44, 87, 88]. Fatigue in caregivers caused by shift work can have drastic consequences for both workers and patients. Education on the hazards and causes of fatigue must therefore be promoted for all care providers.

As described above, coping strategies to prevent fatigue and insomnia may include scheduled napping, exposure to light at work, and special nutrition guidelines. Especially bright light has proven effective to reduce both fatigue and insomnia [52]. Since there are several possibilities for the use of light in the workplace depending on different shift schedules, there is still a lack of standardized recommendations for light therapy. It is taken for granted that light in the workplace mitigates sleepiness, lifts the mood, and improves the mental status in general. Effective use of light may even lead to an adaptation to the extended night work period and facilitate the subsequent re-adaptation to daytime life [89]. The outlined benefits of bright light in industrial settings should be used widely to enhance the adaptation of the human body to night work. Future research should future research should canvass the genetic predisposition for maladaptive circadian phase in night shift workers.

### Expert recommendation

Each company that employs shift workers should generally provide knowledge and support and implement coping strategies against fatigue and insomnia. Since there is a wide range of different rotating shift systems, these measures must be adapted individually in every company. The implementation of exposure to bright light is an effective measure, since it can be easily put into daily practice.

Companies observing these recommendations for their shift working employees in the future would take a major step towards health protection and safety in the work place. We state that companies employing shift workers should ensure optimal environmental and basic conditions: a silent dark room as an opportunity for a nap, a pleasant and undisturbed surrounding for a healthy meal including plenty of protein and carbohydrates, and bright light devices. CBT for insomnia in shift workers could be effective but additional studies are needed in this regard. Moreover, individual chronotypes should be taken into consideration in the allocation of shifts. An optimal approach would be an individually adjusted strategy, which is an elaborate process and has not been examined yet. Our present review aims to give a summary of general recommendations for coping strategies that have been tested and are easy to apply in daily practice.

## Abbreviations

CBT, cognitive-behavioral therapy; MCTQShift, Munich chronotype questionnaire for shift workers; SSI, standard shift work index; SWD, shift work disorder
